# The proteasome: A key modulator of nervous system function, brain aging, and neurodegenerative disease

**DOI:** 10.3389/fcell.2023.1124907

**Published:** 2023-04-13

**Authors:** Kanisa Davidson, Andrew M. Pickering

**Affiliations:** ^1^ Department of Psychology, University of Alabama at Birmingham, Birmingham, AL, United States; ^2^ Center for Neurodegeneration and Experimental Therapeutics (CNET), Department of Neurology, Heersink School of Medicine, University of Alabama at Birmingham, Birmingham, AL, United States

**Keywords:** proteasome, immunoproteasome, synaptic plasticity, Alzheimer’s disease, brain aging, ubiquitin proteasomal system, Parkinson's disease, huntington’s disease

## Abstract

The proteasome is a large multi-subunit protease responsible for the degradation and removal of oxidized, misfolded, and polyubiquitinated proteins. The proteasome plays critical roles in nervous system processes. This includes maintenance of cellular homeostasis in neurons. It also includes roles in long-term potentiation via modulation of CREB signaling. The proteasome also possesses roles in promoting dendritic spine growth driven by proteasome localization to the dendritic spines in an NMDA/CaMKIIα dependent manner. Proteasome inhibition experiments in varied organisms has been shown to impact memory, consolidation, recollection and extinction. The proteasome has been further shown to impact circadian rhythm through modulation of a range of ‘clock’ genes, and glial function. Proteasome function is impaired as a consequence both of aging and neurodegenerative diseases. Many studies have demonstrated an impairment in 26S proteasome function in the brain and other tissues as a consequence of age, driven by a disassembly of 26S proteasome in favor of 20S proteasome. Some studies also show proteasome augmentation to correct age-related deficits. In amyotrophic lateral sclerosis Alzheimer’s, Parkinson’s and Huntington’s disease proteasome function is impaired through distinct mechanisms with impacts on disease susceptibility and progression. Age and neurodegenerative-related deficits in the function of the constitutive proteasome are often also accompanied by an increase in an alternative form of proteasome called the immunoproteasome. This article discusses the critical role of the proteasome in the nervous system. We then describe how proteasome dysfunction contributes to brain aging and neurodegenerative disease.

## 1 Introduction

The proteasome is a dynamic intracellular complex necessary for protein turnover and degradation of damaged proteins. It is the key holoenzyme of the ubiquitin proteasome system, involved in the regulation of proteins and maintenance of cellular signaling (Hershko, et al., 1980).

**FIGURE 1:**
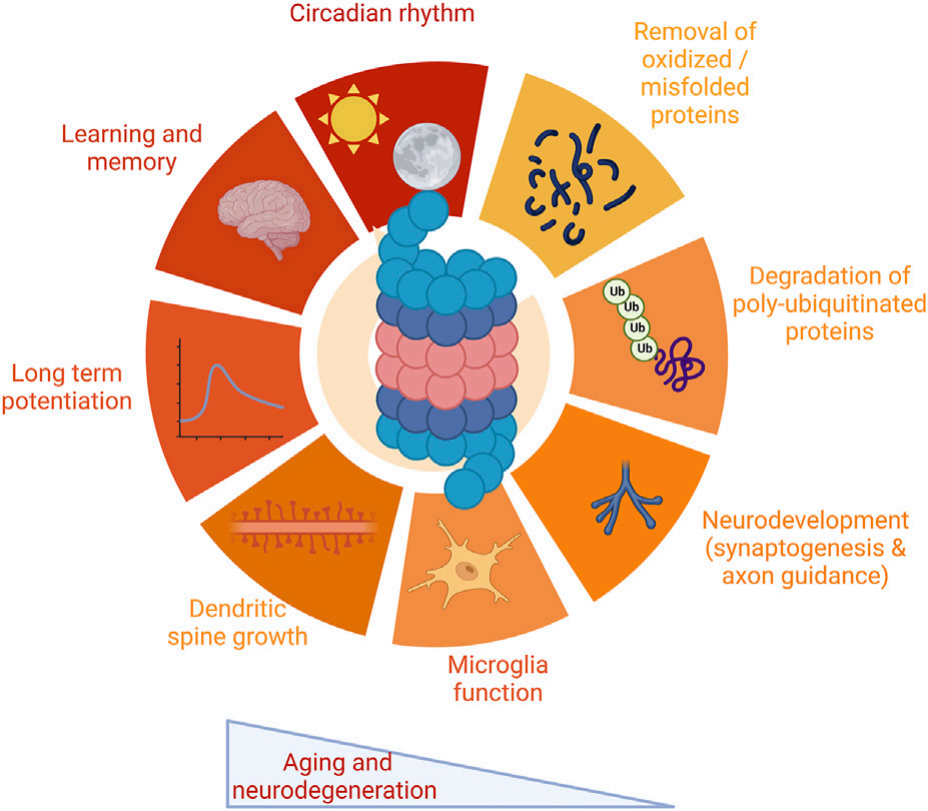
Multiple functions of the proteasome in the nervous system. The proteasome plays critical roles in a wide range of functions critical to the nervous system. These include protein degradation, both of oxidized and misfolded proteins as well as degradation of polyubiquitinated proteins. The proteasome impacts a range of functions specific to the nervous system, including synaptic physiology and plasticity, neurodevelopment and glial cell function, and maintenance of circadian rhythm. The proteasome modulates learning and memory. Proteasome impairment is a robust feature both of aging and neurodegenerative disease, leading to dysfunction in these pathways. Images Created with BioRender.com.

In the nervous system, the proteasome is important for protein degradation and maintenance of cellular homeostasis in neurons, glial cells, and brain health. Furthermore, a growing body of work suggests that the proteasome has key neuron-specific roles, particularly in long-term facilitation (Upadhya et al., 2004) and potentiation (Dong et al., 2008), dendritic spine growth (Hamilton et al., 2012), and neurodevelopment (Hamilton and Zito, 2013; Costa et al., 2019). In addition, the proteasome is involved in synaptic plasticity and protein turnover necessary for learning and memory formation (Lopez-Salon et al., 2001). The proteasome also is a factor in the regulation of clock proteins in the nervous system, impacting circadian rhythm (Ceriani et al., 1999; Grima et al., 2002; Eide et al., 2005). These topics are discussed in detail in Section 2 and summarized in Figure 1.

The function of the proteasome decreases with aging in many tissues, including in the nervous system (Keller et al., 2000a; Keller et al., 2000b). This decline impacts many of the above-described critical nervous system processes necessary for brain health and may contribute to age-related cognitive declines. On the other hand, augmentation of the proteasome system appears to reduce age-related cognitive deficits (Munkacsy et al., 2019). This topic is discussed in detail in Section 3. Moreover, increasing evidence suggests that impairment of the proteasome contributes to progression in Alzheimer’s disease (Keller et al., 2000a; Keller et al., 2000b; Shringarpure et al., 2000; Almeida et al., 2006; Rosen et al., 2010; Chocron et al., 2022), Parkinson’s disease (Zhou et al., 2004; Betarbet et al., 2006; Wang et al., 2006; Zeng et al., 2006; Sun et al., 2007), amyotrophic lateral sclerosis (Kabashi et al., 2012), and Huntington’s disease (Seo et al., 2004). Furthermore, a compensatory induction of the immunoproteasome (an alternative form of the proteasome) is also observed in both brain aging and neurodegenerative disease, although it is unclear if this induction is protective or detrimental (Diaz-Hernandez et al., 2003; Gavilan et al., 2009). These topics are discussed in detail in Section 4.

In this review, we will highlight current perspectives on the proteasome and ubiquitin-mediated functions in brain aging, learning, memory, and neurodegenerative disease.

## 2 Proteasome forms and function

### 2.1 The 20S proteasome

**FIGURE 2:**
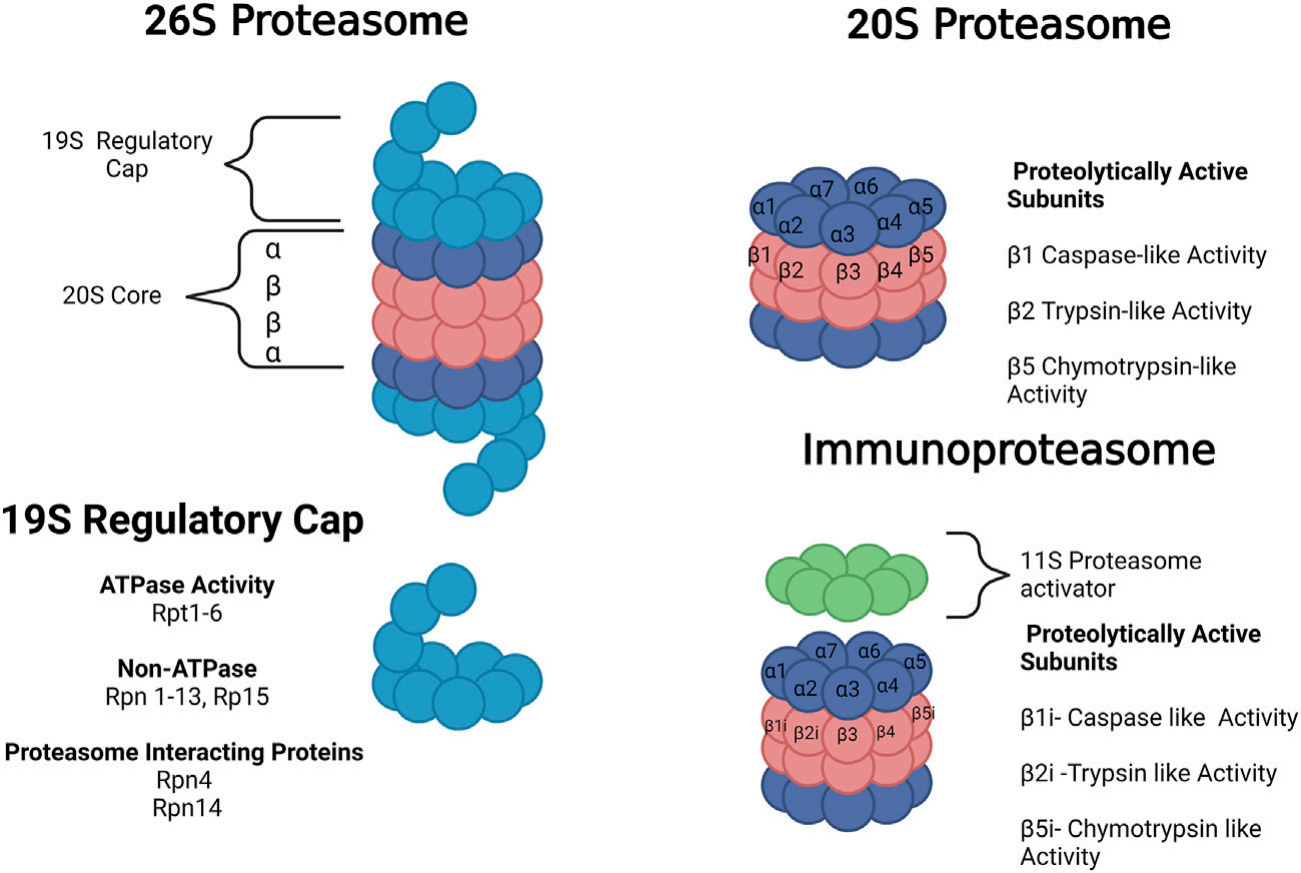
Forms of the proteasome. The 20S proteasome represents free 20S. The 26 S proteasome is composed of the 20S core with a 19S regulatory cap at the top or bottom of the core. The immunoproteasome contains a 20S core with 3 alternative subunits (β1i, β2i and β5i) and an 11S regulatory cap. The 20S proteasome core is composed of 28 subunits divided into 4 heptameric rings in an α ring, β ring, β ring, α ring conformation composed of subunits α1-7 or β1-7, respectively. There are 3 proteolytically active subunits in the core–β1, β2, and β5–associated with caspase-like, trypsin-like, and chymotrypsin-like activities, respectively. The 19S regulator is composed of a range of subunits with ATPase, non-ATPase, and proteasome-interacting functions. Images Created with BioRender.com.

The 20S proteasome is a barrel-shaped protein composed of four heptameric rings. The rings have an αββα configuration of one 7-subunit alpha ring, two 7-subunit beta rings, and one 7-subunit alpha ring. The alpha rings are the antechamber. The inner beta rings are the catalytic chamber of the 20S core. Target proteins are unfolded at the alpha-ring and fed into the barrel, where the peptide is cleaved by proteolytically active subunits on the β-ring. These include the B1 (*PSMB1* or Y), B2 (*PSMB2* or Z), and B5 (*PSMB5* or X) subunits which produce chymotrypsin-like, trypsin-like, and caspase-like activities, respectively. These enzymatic-like activities can cleave hydrophobic, basic, and acidic residues (Figure 2). The 20S proteasome is capable of selective degradation of oxidized or misfolded proteins. It is thought the targeted protein’s exposed hydrophobic residues will selectively bind to the alpha ring, facilitating their unfolding into the core for degradation (Pickering and Davies, 2012). More recent work has also suggested that the 20S proteasome itself may be capable of degrading polyubiquitinated proteins (Sahu and Glickman, 2021).

### 2.2 The 26S proteasome

The 26S proteasome is composed of one or two 19S regulatory caps bound to the top or bottom alpha rings of the 20S proteasome, as shown in Figure 2 (Collins and Goldberg, 2017). Unlike the 20S proteasome, the 26S proteosome shows no preference for oxidized proteins but instead will selectively degrade polyubiquitinated proteins in an ATP-dependent manner (Davies, 2001; Shringarpure et al., 2001). The 19S regulatory cap will selectively bind to proteins carrying a polyubiquitin tag, unfolding the protein and feeding it into the 20S core. The 19S cap contains multiple subunits, including Rpn1, Rpn10, and Rpn13, which are responsible for the initial ubiquitin binding. Usp6/Usp14 and Uch37 remove ubiquitin and activate the 19S regulatory unit. Rpn11 is also essential for the removal of the ubiquitin chain. The 19S ATPase is an enzyme responsible for committing to the binding of the protein and the proteasome. It is also responsible for unfolding and translocating the protein into the 20S gate opening, which is composed of Rpt2, Rpt3, and Rpt5 (Collins and Goldberg, 2017). In rat cortex, single-capped and free 20S forms each comprise 43% of the total proteosome, with double-capped 26S forms comprising the remaining 14% (Tai et al., 2010). This abundance changes with age, as discussed in Section 3.

### 2.3 The ubiquitin proteasome system

The proteasome is the central catalytic enzyme in the ubiquitin proteasome system. Ubiquitin tags have been well-established in protein turnover and are necessary for the ubiquitin proteasome system to function (Hershko et al., 1980). Through polyubiquitination, a chain of ubiquitin proteins is added to the targeted protein and acts as a marker for degradation by the 26S proteasome. Degradation is initiated by the recognition of a polyubiquitin tag (of at least 4 ubiquitin residues) by the 26S proteasome. Four ubiquitin residues are argued to constitute the minimum length for proteasome recognition (Thrower et al., 2000). But more recent work has suggested the proteasome system is more flexible than previously thought and that the proteasome may process proteins either with monoubiquitin or multiple monoubiquitin residues (Braten et al., 2016). Ubiquitin linkage at lysine 48 (K48) is the dominant form targeted for proteasome degradation. Linkage at lysine 63 (K63) can produce targeting for proteasome degradation (Ohtake et al., 2018) and can also target proteins for degeneration through autophagy (Dosa and Csizmadia, 2022). Like the proteasome system, the autophagy system uses ubiquitination as a signal. This topic is discussed in detail in (Kirkin et al., 2009).

For proteasomal recognition of polyubiquitination to occur, the linkage is recognized by 19S regulatory cap subunits Rpn13, Rp1, Rpn10, and Rpn11. These different subunits determine the type of binding by the complex’s four ATPase subunits. This is a selective and efficient process needed for committing to the degradation or reuse of the targeted protein (Collins and Goldberg, 2017). Ubiquitin linkage can also occur at a number of other so-called non-canonical sites on ubiquitin, including M1, K6, K11, K27, K29, and K33. Such sites can occur on their own or in concert with K48/K63 and are involved in a broad range of different cellular processes. This topic is reviewed in detail in (Tracz and Bialek, 2021).

**FIGURE 3:**
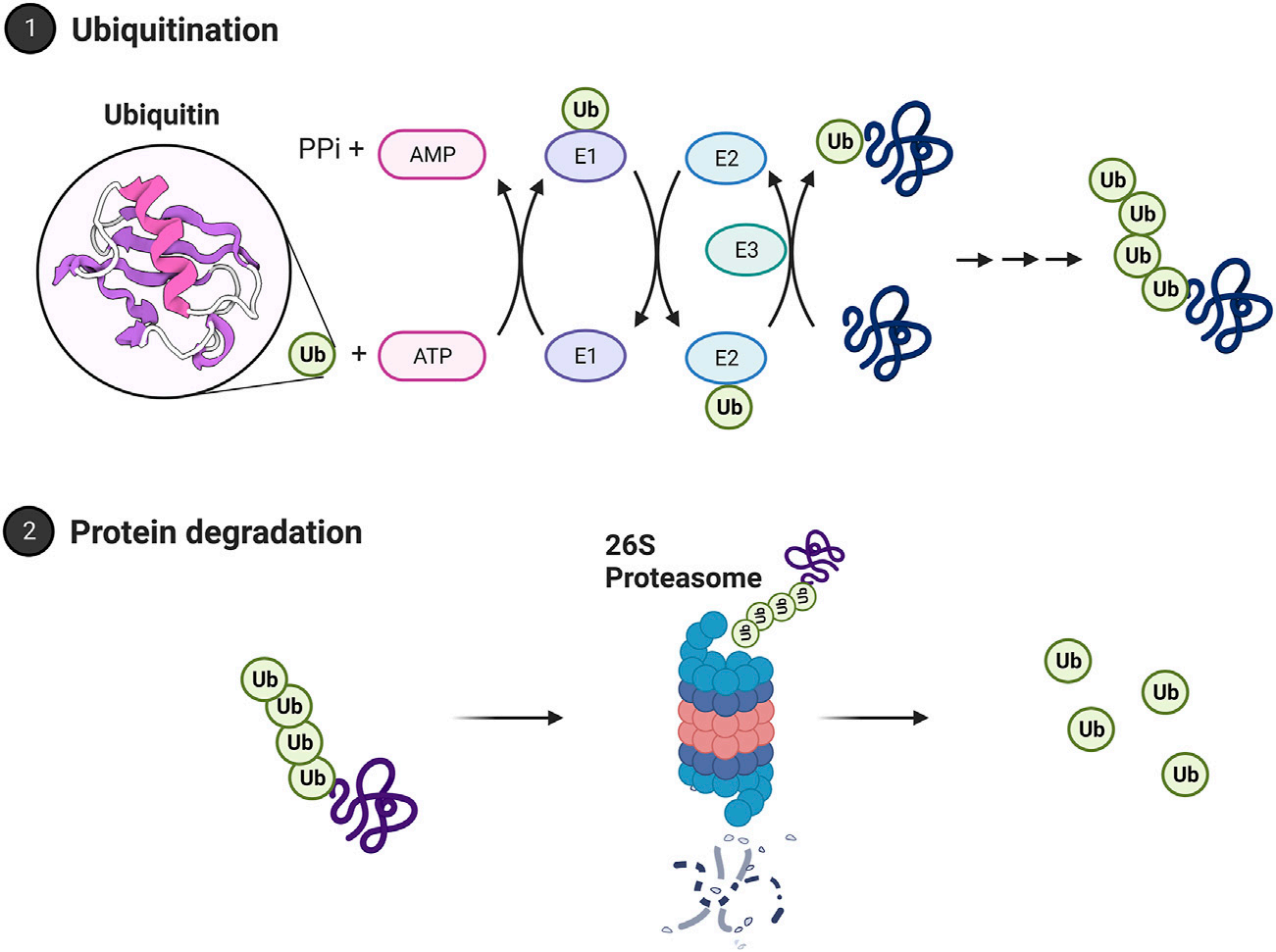
The ubiquitin proteasome system. Proteins are polyubiquitinated in a process relying on E1, E2, and E3 ubiquitin ligases. Ubiquitin is bound to the E1 activating enzyme in an ATP-dependent process. The E1 ubiquitin activating enzyme will bind to and transfer ubiquitin to an E2 ubiquitin conjugating enzyme. An E3 ubiquitin ligase will then bind both the ubiquitinated E2 enzyme and the protein target, facilitating the transfer of ubiquitin to the target protein. A chain of ubiquitin residues can form on a target protein through this approach. A chain of at least 4 ubiquitin residues may be targeted to the 26S proteasome for degradation. The polyubiquitin chain is bound by the 19S regulatory cap and the protein fed into the core for degradation. Ubiquitin is then recycled by deubiquitinating enzymes. Images Created with BioRender.com.

Selective targeting of protein for the ubiquitin proteasome system is driven by the ubiquitin cascade. This pathway includes three enzymes that act as a cascade system, as shown in Figure 3. It begins when ubiquitin is bound to the C-terminal glycine residue of the E1 enzyme. The E1 enzyme initiates this cascade through ATP hydrolysis to AMP. This results in a thioester bond with ubiquitin. After this activation, the ubiquitin carrier protein, E2, moves ubiquitin from E1 to E3, the ubiquitin ligase protein. E3 is the final protein that binds ubiquitin to the protein at the lysine residue (Ciechanover, 1998). In mammals, there are two E1 enzymes, tens of E2 ubiquitin conjugating enzymes, and hundreds of E3 ubiquitin ligases. This redundancy allows for greater specificity in targeting proteins. E1 is the least regulated, while there are more interactions with E2 and E3 enzymes requiring more specificity (Nagy and Dikic, 2010). Prior work has identified two major E3 enzyme classes. The classes are homologous to the E6-AP C terminus (HECT), and a Really Interesting New Gene finger (RING). The HECTs C terminal region of E6-AP can interact with E2 UbH5 and UbH7. This discovery led to recognition of the HECT domain, which in the nervous system is regulated by intracellular calcium and assists with the translocation of receptors to the plasma membrane (Glickman and Ciechanover, 2002; Hegde, 2004; Hegde, 2010). RING finger E3 has seven cysteine residues and a histamine residue, creating a folded domain bound to two zinc ions. They do not form a thioester bond with ubiquitin and have two categories within themselves. Single subunit ring fingers have a domain that is recognized in the substrate in the same protein. This structure has been studied in the ubiquitination of postsynaptic density protein PSD-95 and in parkin associated with Parkinson’s disease (Kitada et al., 1998; Colledge et al., 2003). Multi-subunit RING finger E3s subunits are the SKP1-cullin-F-box protein complex and anaphase promoting complex.

### 2.4 The immunoproteasome

The immunoproteasome was discovered in the late 1990s as an alternative and inducible form of the proteasome. The key role of the immunoproteasome and the reason for its name is its role in the generation of peptides for major histocompatibility complex I (MHC-1) antigen presentation, a role also performed by the 20S/26S proteasome (McCarthy and Weinberg, 2015). The immunoproteasome appears at least as capable as the 20S proteasome in selective degradation of oxidized proteins and may be induced as part of the cell’s oxidative stress response (Pickering et al., 2010). The immunoproteasome structure has three alternative subunits (β1i, β2i, and β5i), replacing the constitutive (non-induced) proteasome (β1, β2, and β5 subunits, respectively) (Figure 1). The immunoproteasome alters cleavage patterns to generate peptides with hydrophobic C terminals that are to be presented in the MHC-1.

The immunoproteasome subunits are regulated by interferon-gamma (INF-γ), making expression of the immunoproteasome inducible under both immune stress and oxidative insult (Pickering et al., 2010; Angeles et al., 2012). The PA28 alpha-beta (11S regulator) can form a component of the immunoproteasome. It is also upregulated by INF-γ (Tanaka and Kasahara, 1998). Some work suggests the immunoproteasome has increased chymotrypsin-like activity (Seifert et al., 2010), but reduced caspase-like activity (Tanaka, 2009). However, other work suggests the immunoproteasome and constitutive proteasome do not differ in activities (Nathan et al., 2013). β5i has chymotrypsin activity and hydrophilic properties to assist with the C terminal of hydrophobic proteins. These peptides are then presented to the MHC-1 molecule to regulate immune response and signaling (Groettrup et al., 2010).

A key function of the immunoproteasome is MHC-1 antigen presentation. The MHC is a polygenic complex with different molecules that bind to different peptides for a specific immune response (Mantel et al., 2022). This pathway occurs in antigen-presenting cells and uses peptides from proteolytic degradation as a means of ‘sampling’ the proteome. The immunoproteasome localized around the endoplasmic reticulum (ER) will cleave proteins which are then translocated to the ER through the TAP channel and incorporated into MHC-1 complexes for antigen presentation on the plasma membrane for screening by T cells.

The immunoproteasome in the nervous system appears to be expressed both in immune and non-immune cells in the brain, including in astrocytes, bone marrow-derived immune cells, oligodendrocytes, and Purkinje cells. Immunoproteasome expression is low in these regions but is sharply elevated upon injury (Ferrington et al., 2008). Immunoproteasome expression is also elevated under conditions of aging and neurodegenerative disease, which will be discussed in Sections 3, 4, respectively.

### 2.5 Plasma membrane proteasome

The nervous system appears to possess an enrichment for proteasome localized to the plasma membrane, which is not observed in cells derived outside the nervous system. In mouse hippocampal slices and human neuronal tissue, cryo-electron microscopy analyses show that approximately 40% of proteasome (specifically 20S proteasome) had a plasma membrane localization, which was not observed in non-neuronal cell cultures. Plasma membrane proteasome appears to have one end exposed to the extracellular space and can be inhibited by cell-impermeable proteasome inhibitors (Ramachandran and Margolis, 2017). It appears that plasma membrane proteasome can span the plasma membrane space and degrade intercellular proteins, releasing material into the extracellular space. Furthermore, inhibition of the plasma membrane proteasome impairs calcium signaling, thus affecting key neuronal processes in neuronal function (Ramachandran and Margolis, 2017), a topic expanded on in Section 2. There is also evidence that the plasma membrane proteasome has a number of specialized regulatory roles. For example, plasma membrane proteasome appears to degrade a number of ribosome-associated nascent polypeptides synthesized during neuronal stimulation, as well as factors involved in response to extracellular signaling and neuronal circuitry such as Npas4 and c-fos (Ramachandran et al., 2018).

## 3 Proteasomal impact on key nervous system processes

### 3.1 Proteasome regulation of long-term potentiation and long-term facilitation

Long-term potentiation (LTP) and long-term facilitation (LTF) refer to the long-term strengthening of synapses between neurons in response to repetitive stimuli. These processes are important in learning and memory, including memory consolidation and/or retrieval (Cooke and Bliss, 2006).

A number of studies suggest a critical role for the proteasome system in formation and maintenance of LTF. Proteasome function is enhanced under LTF, leading to enhanced degradation of proteins that inhibit LTF (Chain et al., 1999; Upadhya et al., 2004). In *Aplysia* (sea slugs), long-term defensive reflexes are driven by cAMP-dependent ion-channel regulation caused by selective loss of the R-subunit of phosphorylated kinase A (PKA). PKA is a tetramer composed of one R unit (regulatory subunit) and 2 C units (catalytic subunits). Declines in the R subunit are thought to result in free C subunits, which may promote LTF (Soberg and Skalhegg, 2018). This process appears not to be driven by transcriptional changes, but is thought to be regulated by degradation of the R subunit by the ubiquitin proteasome system (Greenberg et al., 1987; Hegde et al., 1993). In support of this concept, the proteasome inhibitor lactacystin blocks (5-HT induced) LTF, which may then be rescued by co-administration of free C subunits (Chain et al., 1999). Furthermore, it appears that long-term facilitation results in an upregulation of a neuron-specific ubiquitin C-terminal hydrolase (Ap-uch) by serotonin signaling, which interacts with the proteasome and elevates proteasome activity (Hegde et al., 1997). Induction of LTF also results in enhanced ubiquitination and degradation of CREB1b, a repressor of the neuronal plasticity transcription factor CREB (Upadhya et al., 2004).

**FIGURE 4:**
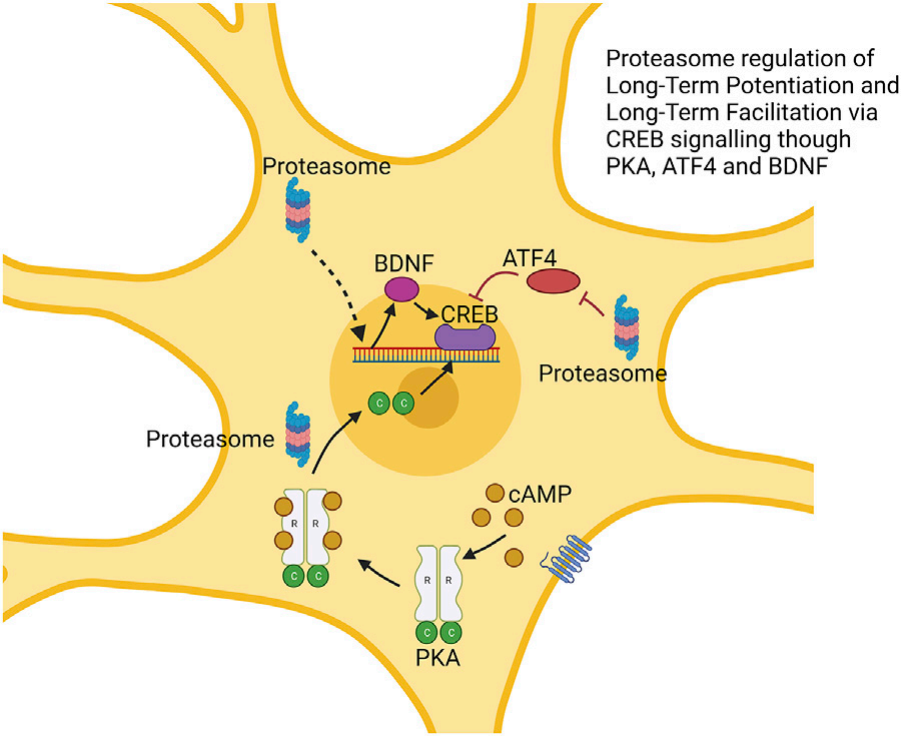
Proteasome modulation of long-term potentiation via regulation of CREB signaling. The proteasome regulates multiple factors impacting CREB function. This includes degradation of CREB repressor ATF4. cAMP-induced degradation of the R subunit of protein kinase alpha (PKA) produces free C subunit, which may enhance CREB function. Augmented BDNF expression also enhances CREB function. → is intended to denote causation. ┴ is intended to denote inhibition. ⇢ is intendend to denote causation through a mechanism that is not fully defined. Images Created with BioRender.com.

In vertebrate studies of LTP, the proteasome has a more complex biphasic role. Treatment of rat hippocampal slices with the proteasome inhibitor lactacystin impaired LTP which was rescued under co-treatment with protein translation inhibitors (Fonseca et al., 2006). Separately, the proteasome inhibitors β-lactone and epoxomycin produced a greater initial (early LTP) response but reduced (late LTP) maintenance (Dong et al., 2008). Proteasome inhibition appears to repress degradation of ATF4 (a repressor of CREB-mediated transcription) and to block transcription of BDNF, another regulator of CREB. Thus, the proteasome’s role in LTP maintenance occurs through the degradation of CREB repressors, similar to LTF (Dong et al., 2008). The role of proteasome repression in increasing early LTP seems to be driven by processes independent of the nucleus as shown using cell bodies surgically cut and to be prevented by rapamycin which blocks the translation of a range of mRNAs. It is hypothesized that impacts on early LTP stems from the temporary stabilization of proteins that are locally translated (Dong et al., 2008; Hegde, 2010). These processes are shown in Figure 4.

Neuronal activity also seems to cause translocation of the proteasome to the synapse, thereby enhancing the local proteasome activity (Bingol and Schuman, 2006). This process is mediated through the NMDAR receptor. Repression of specific NMDAR subunits reduces the localization of the proteasome to the synapse (Ferreira et al., 2021).

### 3.2 Proteasome modulation of dendritic spine growth

The proteasome also helps to facilitate synaptic strengthening by promoting dendrite growth and density. For example, the proteasome plays a role in dendritic spine outgrowth. Proteasome activity around the dendrites is elevated under synaptic stimulation, the product (in part) of localized redistribution of proteasomes from the dendritic shaft to the synapse spines following synaptic stimulation through an NMDA receptor-regulated pathway (Bingol and Schuman, 2006). In this circumstance, CaMKIIα phosphorylated by the NMDA receptor acts as a scaffold to bind the proteasome and sequester it in the dendritic spines under synaptic activation (Bingol et al., 2010). Other reports show that recruitment of the proteasome to the dendritic spines depends on the NAC1 transcription factor, and that deletion of this gene prevents bicuculline, which enhances synaptic activity, from enhancing proteasome recruitment to the dendritic spines (Shen et al., 2007).

**FIGURE 5:**
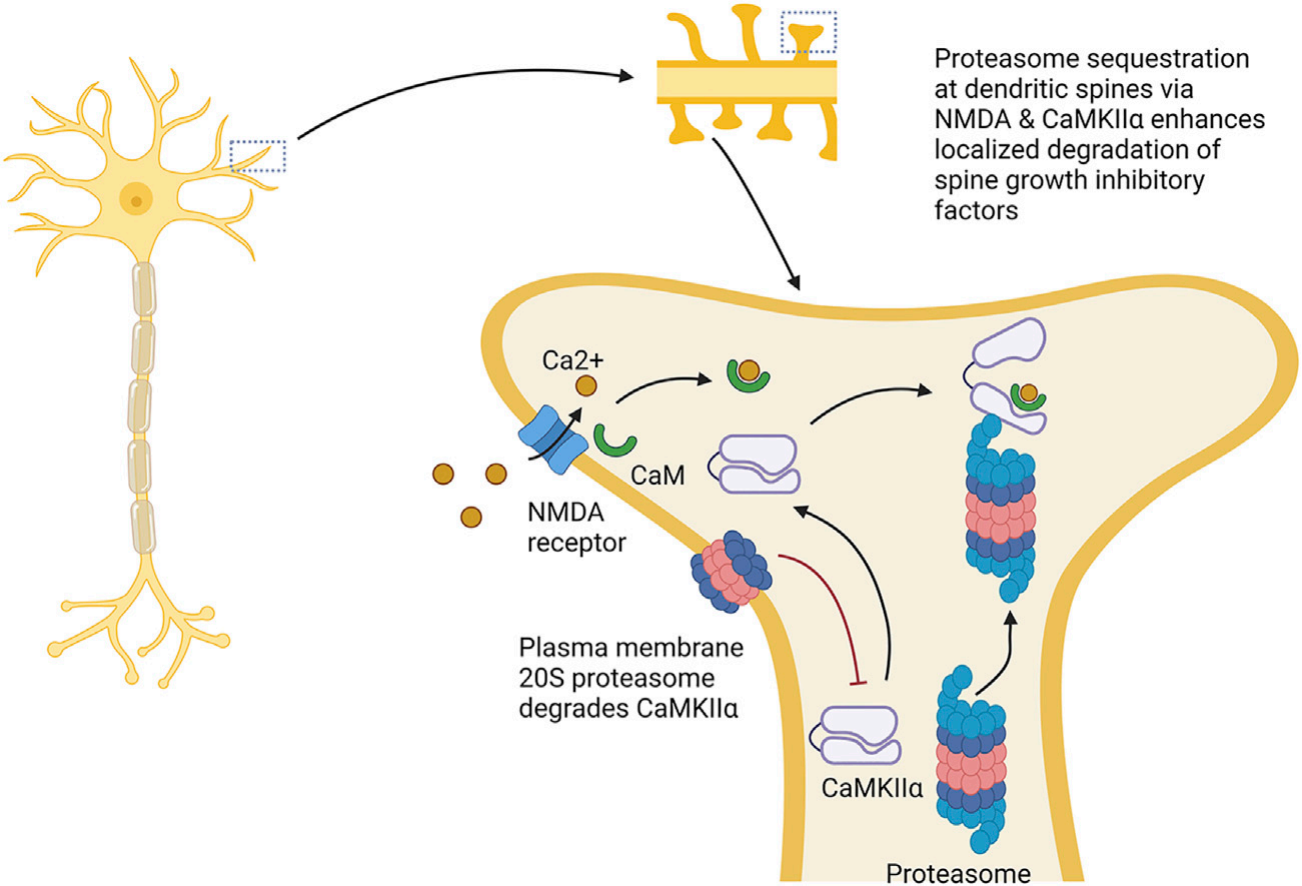
Proteasome modulation of dendritic spine growth. The proteasome plays key roles in dendritic sprine growth. It is thought that this occurs by Ca2+ influx through NMDA receptors, which localizes CaMKIIα to dendritic spines, resulting in the recruitment and activation of the proteasome in dendritic spines. Activation and localization of proteasome to the dendritic spines is thought to promote dendritic spine growth by increasing degradation of factors repressing spine growth. This process is counteracted by the plasma membrane 20S proteasome, which appears to degrade CaMKIIα. → is intended to denote causation. ┴ is intended to denote inhibition. Images Created with BioRender.com.

Treatment with the proteasome inhibitors MG132 or lactacystin both slowed dendritic spine growth, and under proteasome inhibition, bicuculline did not promote dendritic spine outgrowth, suggesting that activity-dependent promotion of dendritic spine growth is proteasome-dependent (Hamilton et al., 2012). This effect seems to depend on the serine120 site on the proteasome subunit RPT6 and CaMKII (Hamilton et al., 2012). In addition, phosphorylation of RPT6 serine120 by CaMKII promotes enhanced proteasome activity (Bingol et al., 2010). As such, CaMKII serves not only to anchor the proteasome to dendritic spines, but also increases proteasome activity in them (see Figure 5).

The somewhat counter-intuitive idea that proteasome augmentation promotes dendritic spine growth has been explained as the consequence of the proteasome’s increased degradation of inhibitory factors that block spine outgrowth (Hamilton et al., 2012). In support of this idea, Ephexin5, which acts as a repressor of spine growth and is a target for proteasome degradation, is reduced following treatment with bicuculline; furthermore, the decline in Ephexin5 is prevented by lactacystin (Hamilton et al., 2017). However, proteasome inhibition appears to repress dendritic spine growth even in an Ephexin5 knockout line. Thus, degradation of Ephexin5 does not seem to be the sole route through which the proteasome promotes dendritic spine growth.

In Section 1.5, we discussed that a novel plasma membrane form of proteasome is found specifically in the nervous system. Recent work has demonstrated a role for this proteasome form in learning and memory. In tadpoles, 33% of proteins generated as a consequence of neuronal signaling were degraded by the plasma membrane proteasome. CaMKIIα (described above) is a target for degradation by the plasma membrane proteasome, and impairing the plasma membrane proteasome increased CaMKIIα levels. Inhibiting the plasma membrane proteasome increases neuronal activity. As such, the plasma membrane proteasome potentially plays a counteracting role to the role described above. Furthermore, inhibiting plasma membrane proteasome function impaired learning-induced visual avoidance capacity in tadpoles suggesting a role in learning and memory (He et al., 2023).

### 3.3 Maintenance of learning and memory by the proteasome

In Sections 2.1 and 2.2 we describe how the proteasome is important in long-term potentiation and facilitation and dendritic spine growth. The proteasome also plays a role in the formation of long-term memory in animal studies. For example, bilateral infusion of lactacystin to the CA1 region of the hippocampus produced retrograde amnesia against an inhibitory-avoidance learning test when given 1–7 h after training but not 10 h after training (Lopez-Salon et al., 2001). Similarly, infusion with the proteasome inhibitor clasto-lactacystin-β-lactone prevented strengthening of memory. When rats were given 2 days of conditioning to a fear-conditioning assay and then treated with a proteasome inhibitor shortly before the second conditioning trial, further conditioning improvements were not observed. Likewise, MG132 impaired the formation of long-term but not short-term taste memory in crabs (Rodriguez-Ortiz et al., 2011). In a similar test of spatial learning and memory, mice were trained in a Morris water maze and then received a proteasome inhibitor after the final trial. The drug reduced immediate crossing of the target location in mice, but not 3 h after the trial (Artinian et al., 2008). These data suggest that the proteasome is involved in the formation of spatial and contextual memory.

The proteasome also appears to play a role in memory retrieval. When fear-conditioned mice were re-exposed to a fear-conditioning context the following day after training, polyubiquitinated proteins declined, presumably from increased proteasome function. As part of this decline in polyubiquitinated proteins, the post-synaptic proteins GKAP and Shank, previously reported as important in long-term potentiation, were rapidly degraded (Lee et al., 2008). It also appears that repression of protein synthesis impairs retrieval while proteasome inhibitors restore memory retrieval (Lee, 2008). This result suggests that memory retrieval is regulated through a balance between protein synthesis and degradation. Moreover, the proteasome appears to play a critical role in memory extinction. For example, animals subjected to fear conditioning over 2 days and then treated with a proteasome inhibitor had reduced memory extinction on the third day (Lee et al., 2008). Similarly, in honey bees, MG132 reduced extinction of conditioned response to an odor paired with sucrose (Felsenberg et al., 2012). Further discussion on the role of the proteasome in memory processes may be found in (Lip et al., 2017).

### 3.4 Control of circadian rhythm by the proteasome

Circadian clocks are involved in a variety of organismal functions from behavior to metabolism. The system is controlled by a set of ‘master’ clock neurons in the suprachiasmatic nucleus of the brain, which entrain peripheral cell-autonomous clocks throughout the body. Circadian rhythm in these cells is driven by the cyclical expression of a number of clock genes, including CLOCK (CLK) and BMAL1 (CYC in *Drosophila*). These proteins are transcriptional regulators of genes involved in circadian rhythm output. CLK and BMAL1 are negatively regulated by the Period and Cryptochrome genes (PER/TIM in *Drosophila*), which repress CLK/BMAL1 activity (Lowrey and Takahashi, 2000; Reppert and Weaver, 2002).

The periodic expression of many clock proteins is heavily reliant on the proteasome system. Thus, impaired proteasome function results in altered or impaired circadian rhythm. In line with this concept, treatment of mammalian fibroblasts with MG132 increased period length (Eide et al., 2005) and MG132 treatment arrested circadian gene expression profiles in a plant cell line (van Ooijen et al., 2011) Similarly, in *Drosophila*, treatment with MG132 arrested oscillation in expression of the TIM and CRY genes (Koh et al., 2006).

The cyclical nature of many proteins involved in circadian rhythm is produced in part by cyclical protein degradation. Proteins are polyubiquitinated in a rhythmic fashion to facilitate rhythmic oscillation in their expression. Repression of a number of E3 ligases impairs circadian rhythm. For example, mutation of the Cullin-RING E3 ubiquitin ligase SLMB impairs the ability to maintain a light-dark cycle in flies under constant dark conditions (Grima et al., 2002). This ligase plays a role in ubiquitination of PER. However, rhythmicity seems to not be driven by changes in levels of the ligase but instead by periodic phosphorylation of PER (Grima et al., 2002). Similarly, mutation of the Cullin-RING E3 ubiquitin ligase JET produces arrhythmic behavior in flies under constant light. This effect appears to be mediated by ubiquitination of TIM. Interactions between TIM and Jet also appear to be regulated by CRY (Ceriani et al., 1999). The HECT E3 ubiquitin ligase CTRIP represents another key modulator of clock genes. This ligase regulates CLK, and knockdown of CTRIP results in permanently high levels of CLK with periodicity (Lamaze et al., 2011). In mammals, the ubiquitin ligase adapter protein β-TrCP is recruited to PER2 through CKIɛ-mediated phosphorylation, driving its turnover (Eide et al., 2005). In all these reports, changes in the level of ubiquitination are brought about by changes in the ligase’s access to the target protein (Ceriani et al., 1999; Eide et al., 2005).

### 3.5 Proteasome regulation of glial cell function

Astrocytes and microglia are essential in regulating the brain’s immune system and modulating synaptic transmission (Verkhratsky and Nedergaard, 2018). Astrocytes are the most abundant cell in the brain and are broadly expressed throughout the brain. They have end feet that create gap junctions and contact multiple blood vessels in the blood-brain barrier, where they modulate the permeabilization of molecules and neurotransmitters necessary for brain health and function. Astrocytes facilitate synaptic transmission by modulating the release of gliotransmitters such as ATP, GABA, glutamate, and D-serine, and monitor the release of neurotransmitters (Chung et al., 2015).

Ubiquitin proteasome activity appears higher in cultured glia compared to cultured neurons as measured by a GFPu reporter construct, with similar differences noted in mouse brains. This is potentially linked with a higher vulnerability to protein aggregate accumulation and cell death in neurons compared to glia (Tydlacka et al., 2008). Furthermore, proteasome inhibition in glia produces a less pronounced increase in nucleic acid oxidation compared to neurons. These data suggest a higher dependency on proteasome function for the removal of oxidized proteins in neurons compared to glia, or possibly a higher oxidative load requiring proteasome removal in neurons (Ding et al., 2004).

There is evidence that proteasome dysfunction is closely correlated with the formation of intermediate filaments such as GFAP and vinmentin in astrocytes, a process considered a hallmark of astrogliosis (abnormal increase in astrocytes). Accumulation of such filaments is commonly seen in aging and neurodegenerative disease where proteasome function is impaired (Cotrina and Nedergaard, 2002). More extensive discussion of proteasome impairment in aging and disease is provided in Sections 3, 4. In cell culture, treatment of astrocytes with the proteasome inhibitors epoxomcicin, lactacystin, and MG132 results in reduced GFAP mRNA and protein levels, and a less pronounced GFAP filamentous network. These effects appear to be driven either by modulation of GFAP transcription factors by the proteasome or direct regulation of the GFAP promoter (Middeldorp et al., 2009). A similar decline in other intermediate filaments such as vinmentin was also observed. Furthermore, after proteasome inhibition, rats showed an impaired ability for astrogliosis after insertion of a foreign body (Middeldorp et al., 2009). This result suggests that the proteasome is involved in the ability of astrocytes to mount a response to insult though modulation of GFAP and other intermediate filaments. The finding that proteasome function promotes rather than inhibits the capacity for astrogliosis is counter-intuitive, given the body of work showing a link between proteasome impairment in aging and neurodegenerative disease and a concurrent increase in astrogliosis. However, complementary induction of the immunoproteasome in these diseases may underlie these effects (discussed in Section 4).

The immunoproteasome system also plays a key role in glia, where immunoproteasome subunits are induced in microglia in response to stroke, infection, or brain injury (Moritz et al., 2017). This induction appears to have functional relevance. In a rat model of ischemic stroke, siRNA knockdown of immunoproteasome subunits resulted in fewer activated astrocytes and microglia and reduced expression of inflammatory cytokines (Chen et al., 2015). Similarly, proteasome inhibition of human fetal microglia by MG132 (which will impair proteasome and immunoproteasome function) inhibited TNF-α release and blocked NF-κB release. Additionally, immunoproteasome inhibition impaired interferon-γ-induced transcriptome changes and inflammatory response. These data suggest that the immunoproteasome modulates the ability of microglia to mount a response to disease or injury (Moritz et al., 2017).

## 4 Proteasome dysfunction as a modulator of brain aging

### 4.1 Proteasome function declines with age

A decline in proteasome function with age has been reported in human T-lymphocytes and lens cells (Andersson et al., 1999; Ponnappan et al., 1999) and in rat heart, kidney, liver, lung, and muscle (Bardag-Gorce et al., 1999; Keller et al., 2000a; Keller et al., 2000b). In the nervous system, aging leads to reduced proteasome activity in the cortex, cerebellum, and spinal cord, though activity appears unchanged in the cerebellum and brain stem (Keller et al., 2000a; Keller et al., 2000b). Impaired proteasome function with age was also reported in the heads of fruitflies (Munkacsy et al., 2019) and the brains of killifish (Kelmer Sacramento et al., 2020). The decline in proteasome function with age is correlated with increases in oxidized proteins and polyubiquitinated proteins throughout the body, including in the brain (Petropoulos et al., 2000; Levine and Stadtman, 2001; Rai et al., 2021).

### 4.2 Altered proteasome composition with age

The ubiquitin ligase system is less affected by aging (Ciechanover et al., 2000), but many individual ligases are either up- or downregulated during the aging process. By contrast, age-related declines in proteasome function appear to be mostly driven by changes in the proteasome itself. This is in part produced by a functional decline of the system. In one report, proteasome isolated from 17-, 38- and 50-year-old patients appeared progressively less capable of degrading proteolytic substrates (Bulteau et al., 2000). Moreover, proteasome composition changes with age. In whole flies, fly heads, and killifish brains, the 26S proteasome declines with age, unlike the 20S proteasome (Vernace et al., 2007; Tonoki et al., 2009; Munkacsy et al., 2019; Kelmer Sacramento et al., 2020). Native-PAGE immunoblots, native-PAGE activity assays, and fractionation revealed a decline in 26S proteasome assembly and a consequent increase in 20S proteasome assembly, although 20S proteasome activity did not increase (Vernace et al., 2007; Tonoki et al., 2009; Munkacsy et al., 2019; Kelmer Sacramento et al., 2020).

There are a number of potential explanations for this change in proteasome composition as a consequence of age. The 20S proteasome is functionally directed to the removal of oxidized and unfolded proteins, while the 26S proteasome is more heavily linked with the degradation of polyubiquitinated proteins. The 20S and 26S proteasomes exist in a dynamic state, with the 26S proteasome capable of disassembling into the 20S form when oxidative burden is elevated and then subsequently reassembling (Grune et al., 2011). Higher oxidative burden in aging, coupled with reduced proteasome functionality, may facilitate disassembly of the 26S proteasome in favor of the 20S proteasome at the expense of maintenance of the ubiquitin proteasome system. In another study, protein levels of 20S proteasome subunits increased with age, but 19S (cap of the 26S) subunits decreased with age (Kelmer Sacramento et al., 2020).

### 4.3 Proteasome augmentation extends lifespan and reduces age-related cognitive deficits

Modulation of the proteasome subunit PSMB5 appears a viable way to enhance proteasome function. Augmentation of this subunit increases protein expression of other proteasome subunits (α and β subunits but not 19S cap subunits) and whole proteasome assembly, through an auto-regulatory mechanism that is not fully understood (Chondrogianni et al., 2005). Augmentation of this subunit and subsequently increased proteasome function extends lifespan in nematode worms (Chondrogianni et al., 2014; Chondrogianni et al., 2015) and fruit flies (Nguyen et al., 2019), and extends lifespan when overexpression is targeted to the nervous system (Munkacsy et al., 2019). Other interventions to enhance proteasome function have also shown pro-longevity effects; augmentation of the proteasome subunit RPN-6 extends lifespan of nematode worms under conditions of proteotoxic stress (Vilchez et al., 2012). Similarly, RPN-11 overexpression is extended at elevated temperature (29°C) in flies. Furthermore, killifish with faster age-related declines in proteasome function are shorter-lived (Kelmer Sacramento et al., 2020). In connection with the discussion in Section 2 of a role for the proteasome in memory formation, enhancing proteasome assembly reduces age-related deficits in associative learning and memory in fruit flies (Munkacsy et al., 2019).

### 4.4 Increased immunoproteasome presentation in aging brain

Levels of the immunoproteasome subunits (both protein and mRNA) are increased with age in hippocampal tissue from rats (Gavilan et al., 2009) and increased with age in the retina from in mice (Hussong et al., 2010). The immunoproteasome responds to oxidative stress both in cell culture and in nervous system tissue (Ferrington et al., 2008; Pickering et al., 2010), and after traumatic brain injury and microglia inflammatory signaling (Moritz et al., 2017). As a consequence, age-related elevation in the immunoproteasome is likely a consequence of elevated oxidative or inflammatory burden in the aged brain. Age-related elevations in immunoproteasome subunits are also reported in clinical studies, but are significantly less in women compared to men (Kammerl et al., 2021). Additionally, as will be discussed in detail in Section 4, immunoproteasome levels are elevated in many neurodegenerative diseases. These results demonstrate that levels of the immunoproteasome are enriched in conditions of nervous system illness. But it is unclear if these elevated levels are detrimental and contribute to impaired/disease conditions, or if they are an important protective response to damage or injury. In support of the latter idea, the immunoproteasome appears to protect against cell death in the nervous system, since animals deficient in immunoproteasome subunits are more susceptible to oxidative cell death (Hussong et al., 2010). Elevated expression of immunoproteasome subunits is also reported in tissue from the long lived naked mole-rats compared to mice (Rodriguez et al., 2012) and immunoproteasome levels are correlated with lifespan in fibroblasts from different primate species (Pickering et al., 2015). This evidence suggests that elevated immunoproteasome expression is evolutionarily conserved in longer-lived species, possibly to protect against age-related oxidative burden.

## 5 Proteasome dysfunction as a modulator of neurodegenerative disease

Many neurodegenerative diseases develop during aging and result from genetic and environmental factors. Proteasome inhibition appears to be a factor in many neurodegenerative diseases, including Alzheimer’s, Parkinson’s, and Huntington’s disease, as well as amyotrophic lateral sclerosis. There is also a growing body of evidence that proteasome inhibition is a pathologic factor in progression of neurodegenerative disease (see below). Table 1 summarizes results to date regarding impaired proteasome function in different neurodegenerative diseases.

**TABLE 1:** Studies examining proteasome function in patients with neurodegenerative diseases or animal/cell culture models.

Disease	Patients/Model	Impact	References
Alzheimer’s disease (AD)	16 AD patients, 9 control patients	Reduced proteasome activity, hippocampus, parahippocampal gyrus, temporal gyrus and parietal lobe. No change in occipital lobe or cerebellum	Keller et al. (2000a), Keller et al. (2000b)
	SH-SY-5Y cell line	Aβ42 treatment reduces proteasome activity	Rosen et al. (2010)
	Tg2576 APP mutant neuron cell culture	Reduced proteasome activity	Almeida et al. (2006)
	*In vitro* purified proteasome + Aβ	Aβ40 inhibits purified proteasome activity	Shringarpure et al. (2000)
	Control Braak 0-1 (N = 11), and AD patients (Braak 2-4 (N = 12), Braak 5-6 (N = 12)), B103 cells + Aβ42, Elav-GS-GAL4:UAS-AB3-42 and Elav-GS-GAL4; UAS-APP,UAS-BACE1 flies, hAPP(J20) mice brains	Impaired proteasome activity in patients after Braak 2, impaired proteasome activity in cells treated with Aβ, and in AD fly and mouse models. Proteasome overexpression and proteasome agonists reduce AD-like deficits	Chocron et al. (2022)
	Control Braak 1-2 (N = 6), and AD patients Braak 6 (N = 12), *in vitro* proteasome + Tau	Proteasome function impaired in AD patients and in purified proteasome incubated with tau filaments	Keck et al. (2003)
	3xTg-AD mice	Impaired proteasome activity; proteasome inhibition increases Aβ + Tau accumulation	Tseng et al. (2008)
	SK-N-SH cell line	Proteasome inhibitor increases BACE1 and APP C99	Qing et al. (2004)
Parkinson’s disease (PD)	10 PD patients and 7 control patients	Proteasome subunits co-localize with TH-positive cells	Bukhatwa et al. (2010)
	6 PD patients and 6 control patients	Reduced α-subunits in substantia nigra but not striatum	McNaught et al. (2002)
	16 PD patients and 13 control patients	Reduced levels of proteasome subunits and proteasome activity in substantia nigra but not other regions	McNaught et al. (2003)
	10 PD patients 7 control patients	Chymotrypsin-like, trypsin-like and caspase-like activity impaired in substantia nigra	(McNaught and Jenner, 2001)
	3 patients in each of 4 groups: PD, AD, Lewy body dementia, and controls	Proteasome function impairment did not correlate with Lewy body presentation	Tofaris et al. (2003)
	SK-N-MC cell line and rat brains	Treatment with rotenone in cells or infusion in rat brains impairs proteasome function	Betarbet et al. (2006)
	SK-N-SH cell line	Treatment with multiple pesticides impairs proteasome function	Wang et al. (2006)
	Marmosets	Treatment with MPTP impairs proteasome function	Zeng et al. (2006)
Huntington’s disease (HD)	Patients with HD (grade 0–1: n = 8, and grade 3–4: n = 9) and controls (n = 6)	Proteasome activity impaired in striatum, cerebellum and (non-significantly) in the substantia nigra	Seo et al. (2004)
	Mice transgenic for HD exon 1	Proteasome function impaired with mutant HD expression	Jana et al. (2001)
	Sca7 HD mice	Proteasome expression increased with disease progression, proteasome activity unchanged; inverse relationship between nuclear inclusion formation and nucleopathy	Bowman et al. (2005)
	HD94 mice	Increased immunoproteasome subunit expression in HD mice	Diaz-Hernandez et al. (2004)
	HD R6/2 mice	Proteasome activity increased in HD mouse. Proteasome activator REGγ highly expressed. Mutation of REGγ does not impact HD progression	Bett et al. (2006)
Amyotrophic Lateral Sclerosis (ALS)	5 ALS patients, 4 control patients	Proteasome activity impaired in spinal cord but not cerebellum; proteasome subunit composition largely unaltered	Kabashi et al. (2012)
	SOD1G93A transgenic mouse	Proteasome activity unchanged. Reduced expression of constitutive proteasome and increased expression of immunoproteasome. Increased accumulation of polyubiquitinated SOD1	Cheroni et al. (2005)
	SOD1G93A transgenic mouse	Decline in constitutive proteasome assembly precedes increase in immunoproteasome	Kabashi et al. (2008)
	SOD1 ALS mutant cell line	Proteasome inhibition increases mutant SOD1 toxicity	Kitamura et al. (2014)

### 5.1 Alzheimer’s disease

Alzheimer’s Disease (AD) represents the leading cause of dementia, with approximately 500,000 new cases a year, and 50 million people worldwide are living with AD. The disease is marked by progressive declines in cognitive and executive function, with accumulation of β-amyloid plaques and hyperphosphorylated tau as key pathologic markers. Lowered activity of the proteasome is a robust feature of AD, described in animal models of AD and in post-mortem brains from AD patients (Keller et al., 2000a; Keller et al., 2000b; Shringarpure et al., 2000; Almeida et al., 2006; Rosen et al., 2010; Chocron et al., 2022).

In patients with AD, proteasome impairment appears most pronounced in the hippocampus and parahippocampal gyrus, with some impairment in the middle temporal gyri, and inferior parietal lobule, but no impairment in other brain regions. It is thought that impaired proteasome function in AD stems from direct inhibition of the proteasome, by β-amyloid or hyperphosphorylated tau. In cell culture and animal model studies, intracellular β-amyloid and hyper-phosphorylated tau can interact directly with the proteasome and inhibit its activity (Shringarpure et al., 2000; Almeida et al., 2006; Myeku et al., 2016). β-amyloid acts as an allosteric inhibitor, preventing opening of the substrate gate of the 20S core and thus inhibiting activity in 20S proteasome activity and inhibiting 26S proteasome function by preventing feeding of substrates into the 20S core by the 19S regulator (Thibaudeau et al., 2018). In patients with AD, levels of proteasome impairment correlated with levels of β-amyloid (Chocron et al., 2022) and hyperphosphorylated tau (Keck et al., 2003). In addition, in an AD mouse model, Aβ immunotherapy reversed proteasome dysfunction (Tseng et al., 2008).

The decline in proteasome function in AD appears to be countered by increased immunoproteasome subunits, both in mouse models and in patients. The increase also appears to be localized to tissue surrounding plaques and to be predominantly from activated glia (Orre et al., 2013).

There is evidence that the proteasome plays a regulatory role in β-amyloid and tau production. Inhibition of proteasome function results in increased accumulation of β-amyloid and tau in cell culture (Tseng et al., 2008). APP, BACE1, and γ-secretase-activating protein, all components of the β-amyloid synthesis pathway, are targets of proteasome turnover, and treatment with proteasome inhibitors increases levels of BACE1 and GSAP in cell lines (Nunan et al., 2003; Qing et al., 2004; Chu et al., 2015). Proteasome augmentation reduces protein levels of APP and Aβ in mice, fly, and cell culture models of AD (Chocron et al., 2022). Furthermore, artificial augmentation of proteasome function (either through genetic or pharmacologic interventions) can rescue AD-like deficits in mouse, fly, and cell culture models (Chocron et al., 2022).

New data suggest an interplay between the plasma membrane proteasome and apolipoprotein E (APOE), the most prevalent genetic risk factor for AD. In post-mortem studies of AD patient brains, plasma membrane proteasome localization was lower in those with greater expression of APOE4 compared to APOE3, and inhibition of the plasma membrane proteasome increased tau aggregation (Paradise et al., 2022).

### 5.2 Parkinson’s disease

Parkinson’s disease (PD) is characterized by rigidity, bradykinesia, postural instability, and neuropsychiatric and cognitive issues. The key histologic features of the disease are the accumulation of Lewy-body aggregates and loss of dopaminergic neurons in the substantia nigra. Like AD, multiple studies show impairments in proteasome function in PD patients. This includes multiple independent reports of reduced protein levels of 20S proteasome α-subunits, no change in β-subunits, and 19S regulator assemblage in the substantia nigra of patients with PD (Bukhatwa et al., 2010). The decline in 20S proteasome α-subunits is specific to the substantia nigra, with no depletion in other brain regions (McNaught et al., 2002). The decline in proteasome subunits appears associated with lower proteasome activity; some reports show a decline in chymotrypsin-like activity, whereas others show impairment in all 3 proteasome activities (chymotrypsin-like, trypsin-like, and caspase-like) (McNaught and Jenner, 2001; McNaught et al., 2003; Tofaris et al., 2003; Arduino et al., 2011; Chang et al., 2019). However, it is unclear if proteasome dysfunction is a cause or consequence of the disease.

Age is the greatest risk factor for PD and the substantia nigra is particularly vulnerable to age-related proteasome dysfunction, supporting a hypothesis that age-related proteasome dysfunction may increase PD risk (Zeng et al., 2005). Furthermore, although purely correlative, many compounds that increase the risk for PD–including paraquat, rotenone, dieldrin, MPTP, and maneb–also impair proteasome function (Zhou et al., 2004; Betarbet et al., 2006; Wang et al., 2006; Zeng et al., 2006; Sun et al., 2007).

PD may be driven in part by broad-scale proteostatic dysfunction. In support of this concept, Lewy bodies, the key pathologic feature of PD, are enriched for ubiquitinated proteins and both proteasome subunits and proteasome activators accumulate in them (McNaught et al., 2006). However, most of the ubiquitination in Lewy bodies is K63-linked, which typically denotes DNA damage repair, cell signaling, or autophagy as preference to K48-linked ubiquitination which typically represents targeting for proteasome degradation (Alexopoulou et al., 2016).

Treatment with proteasome inhibitors or knockout of proteasome subunits can mimic aspects of PD. For instance, *Caenorhabditis elegans* treated with MG132 experience progressive loss of dopaminergic neurons (Caldwell et al., 2009). Mice with Cre/LoxP-induced depletion of the 26S proteasome subunit PSMC1 show neurodegeneration and the formation of Lewy-like bodies (Bedford et al., 2008). Injection of 6 different proteasome inhibitors in those mice produced parkinsonism with bradykinesia, rigidity, tremor, and an abnormal posture. These deficits were reduced when mice received the dopamine receptor agonist apomorphine (McNaught et al., 2004). However, a separate study failed to produce Parkinsonism after proteasome inhibitor treatment in rats or monkeys (Kordower et al., 2006; Manning-Bog et al., 2006).

### 5.3 Huntington’s disease

Huntington’s disease (HD) is an inherited neurodegenerative disorder driven by the presence of long CAG sequence repeats in the Huntingtin protein, making it prone to aggregation. Huntingtin aggregation leads to progressive neurodegeneration; age of onset and rate of progression are, in part, linked to the length of the CAG repeats. Although less studied than in AD and PD, a number of studies suggest that the proteasome is involved in modulating progress of HD.

As in AD and PD (described above), proteasome function appears to be impaired in HD patients. Reduced proteolytic activity (chymotrypsin-like and caspase-like) was found in the striatum of post-mortem brains of patients with HD, both in early and late stages. This result appears to correlate with an increase in ubiquitin detected in late stages. Some declines are also reported in other brain regions, but less pronounced (Seo et al., 2004). Other studies have reported impaired proteasome function in cell culture models of HD (Jana et al., 2001). However, mouse models of HD (unlike patients) may not experience a decline in proteasome activity, as no change in activity was reported in R6/2 or HD94 mice (Diaz-Hernandez et al., 2003; Bett et al., 2006) This is possibly explained by an induction of the immunoproteasome that may compensate for decreased proteasome activity (Diaz-Hernandez et al., 2003).

It has been postulated that impaired proteasome function is driven by inhibition from filamentous huntingtin. For example, filamentous huntingtin aggregates isolated from a mouse model of HD showed some capacity as a proteasome inhibitor (Ortega et al., 2007).

The link between the proteasome and HD was first put forward with work showing that inclusion bodies (a pathologic hallmark of HD) appear to contain ubiquitin and proteasome subunits (DiFiglia et al., 1997; Cummings et al., 1998). However, these inclusion bodies are not in themselves drivers of proteasome impairment. Furthermore, incubation of inclusion bodies isolated from a mouse model of HD failed to inhibit proteasome activity (Ortega et al., 2007). This sequestration may represent a protective response to Huntingtin toxicity but present as a side effect of proteasome depletion (Ortega et al., 2007).

It has also been hypothesized that the proteasome may enhance the toxicity of huntingtin. The proteasome’s three forms of proteolytic activity (chymotrypsin-like, trypsin-like, and caspase-like) each targets specific sequences for degradation. However, the series of glutamine repeats present in the HD mutation does not represent a target sequence for the proteasome. This was shown experimentally, when a peptide of 10–30 glutamine repeats flanked by lysine residues was cleaved at its flanking sequences but not in the main glutamine sequence (Venkatraman et al., 2004). This result suggests that the proteasome might actually promote formation of toxic aggregation-prone PolyQ fragments (Ortega et al., 2007).

### 5.4 Amyotrophic lateral sclerosis

Amyotrophic lateral sclerosis (ALS) is a progressive and age-related motor neuron degenerative disease. A familial mutation in superoxide dismutase (*SOD1*) is a risk factor of ALS, but most cases occur sporadically. Similar to AD, PD, and HD, there is a reported impairment in proteasome function in ALS. In a study of spinal cords from ALS patients, all three proteasome activities (chymotrypsin-like, trypsin-like, and caspase-like) were impaired, in tandem with a decline in levels of the proteasome β5 subunit (Kabashi et al., 2012). Of note, although decreased proteasomal activity and expression were reported in the ventral and dorsal spinal cord, no significant decline was observed in the cerebellum (Kabashi et al., 2012). Similar findings have been made in models of ALS. In the SOD1-G93A mouse model, which contains a familial SOD1 mutation, the constitutive proteasome decreased (Cheroni et al., 2005), accompanied by a decline in constitutive proteasome assembly and an increase in ubiquitinated proteins (Kabashi et al., 2008). However, no decline in proteasome activity (measured through peptide substrates) was observed due to a compensatory elevation of the immunoproteasome (Cheroni et al., 2005).

Various studies also propose a modulatory role for proteasome dysfunction in ALS progression. It is argued that the elevation in mutant SOD1 in SOD1-G93A mice is independent of transcription, and instead a product of reduced mutant SOD1 degradation as a consequence of proteasome dysfunction. This perspective supports a role for proteasome dysfunction as an accelerating factor in this mouse model of ALS (Cheroni et al., 2005). Furthermore, proteasome dysfunction appears to modulate toxicity effects from mutant SOD1 expressed in human cell culture. Under normal conditions, mutant SOD1 produced very low toxicity effects, but became highly toxic under proteasome inhibition (Kitamura et al., 2014). Additionally, TDP-43 aggregation is associated with ALS progression, and its mutation can cause familial ALS. This process appears to be closely regulated both by the proteasome and autophagy systems. Accumulation of aggregates of ubiquitinated TDP-43 is a hallmark of ALS. This accumulation appears independent of the ubiquitination machinery, but may be produced by treatment with proteasome or autophagy inhibitors. This concept suggests that proteasome dysfunction may contribute to ALS-related accumulation of TDP-43 aggregates (Urushitani et al., 2010).

## 5 Conclusion

The proteasome system plays critical roles in many functions of the nervous system. These include the formation and strengthening of memory through the promotion of long-term potentiation by augmentation of the CREB transcription factor–either by degrading repressors of CREB or subunits of activators, which repress their function (Greenberg et al., 1987; Hegde et al., 1993; Hegde et al., 1997; Chain et al., 1999; Upadhya et al., 2004; Dong et al., 2008; Hegde, 2010; Soberg and Skalhegg, 2018; Ferreira et al., 2021). The proteasome also plays a key role in dendritic spine formation. The proteasome is sequestered in the dendritic spine through signaling by CaMKIIα and the NMDA receptor and is thought to then cause local degradation of factors that repress dendritic spine growth. The result is increased growth of dendritic spines (Bingol and Schuman, 2006; Shen et al., 2007; Bingol et al., 2010; Hamilton et al., 2012; Hamilton et al., 2017). Much of this work has been performed with proteasome inhibitors. Treatment with proteasome inhibitors appears to impair memory consolidation and retrieval. Surprisingly, it also appears to be required for memory extinction and proteasome impairment in animal models (Lopez-Salon et al., 2001; Artinian et al., 2008; Lee, 2008; Lee et al., 2008; Rodriguez-Ortiz et al., 2011; Felsenberg et al., 2012).

In both aging and neurodegenerative diseases, constitutive proteasome function is impaired, although the underlying causes appear distinct. In aging, impaired proteasome function appears to be driven by a decline in 26S assembly in favor of 20S proteasome assembly. This is may be explained either by a regulated shift to higher 20S proteasome capacity to accommodate an increased oxidative load at the expense of ubiquitinated protein turnover, or an impairment in 26S proteasome assembly capacity (Vernace et al., 2007; Tonoki et al., 2009; Munkacsy et al., 2019; Kelmer Sacramento et al., 2020).

A similar impairment in proteasome function is observed in AD, PD, HD, and ALS. Indeed, impaired proteasome function is an almost universal feature of neurodegenerative diseases. However, the mechanism of this impairment is quite distinct from that of aging and is different in each disease. In AD, the proteasome is directly inhibited by β-amyloid or tau (Shringarpure et al., 2000; Almeida et al., 2006). In PD, a decline in proteasome expression and function is also observed specifically in the substantia nigra, although the cause of this decline is less clear. It is also produced by pesticides, which are a PD risk factor (McNaught and Jenner, 2001; McNaught et al., 2003; Tofaris et al., 2003; Arduino et al., 2011; Chang et al., 2019). Proteasome function is impaired in HD patients but is elevated in mouse models (potentially from expression of the immunoproteasome). This impairment in proteasome function seems to stem from active proteasome sequestration. Proteasome sequestration in HD and is potentially a protective response to reduce huntingtin toxicity, as proteasome cleavage of mutant huntingtin can produce a highly aggregate prone degradation product (Jana et al., 2001; Diaz-Hernandez et al., 2004; Seo et al., 2004; Bowman et al., 2005; Bett et al., 2006). In ALS, proteasome function is impaired in patients but not in mouse models; the latter instead have a decline in the constitutive proteasome and a compensatory increase in the immunoproteasome. This change results in an increase in ubiquitinated SOD1. Proteasome impairment has been reported to increase mutant SOD1 toxicity (Cheroni et al., 2005; Kabashi et al., 2008; Kabashi et al., 2012; Kitamura et al., 2014).

In summation, the proteasome plays key roles in many nervous system processes that are impaired as a consequence either of age or neurodegenerative disease. Impairment in these processes may be driven by declines in proteasome function. However, much of the work to date has focused either on correlative work comparing tissue from young vs. old animals/patients, control vs. disease baring patients/animal models, or proteasome inhibition experiments. It has been shown reproducibly that impairment of proteasome function can produce deficits in a wide range of nervous system processes and that proteasome function is impaired both as a consequence of aging and in varied diseases. It has as such been shown that proteasome impairment is *sufficient* to account for deficits either as a consequence of aging or neurodegeneration. However, it has yet to be shown if impairment in proteasome function in aging or neurodegenerative diseases is *necessary* to account for deficits. Such a demonstration would require proteasome augmentation studies to demonstrate that deficits may be corrected through prevention of proteasome dysfunction. Some work has begun to demonstrate this including abilities to rescue cognitive deficits, either as a consequence of aging or neurodegenerative disease (Munkacsy et al., 2019; Chocron et al., 2022) though further investigation is needed.
